# Identification of anti-inflammatory compounds from Zhongjing formulae by knowledge mining and high-content screening in a zebrafish model of inflammatory bowel diseases

**DOI:** 10.1186/s13020-021-00452-z

**Published:** 2021-05-31

**Authors:** Yunru Yu, Jing Chen, Xiaohui Zhang, Yingchao Wang, Shufang Wang, Lu Zhao, Yi Wang

**Affiliations:** 1 Pharmaceutical Informatics Institute, College of Pharmaceutical Sciences, Zhejiang University, Hangzhou, 310058 China; 2 Innovation Institute for Artificial Intelligence in Medicine of Zhejiang University, Hangzhou, 310058 China; 3 State Key Laboratory of Component-Based Chinese Medicine, Tianjin University of Traditional Chinese Medicine, Tianjin, China

**Keywords:** Inflammatory bowel diseases, Zhongjing formulae, Knowledge mining, High-content screening, Zebrafish, Multimodal identification

## Abstract

**Background:**

Inflammatory bowel diseases (IBD) are chronic relapsing intestinal inflammations with increasing global incidence, and new drug development remains in urgent demand for IBD management. To identify effective traditional Chinese medicine (TCM) formulae and compounds in IBD treatment, we innovatively combined the techniques of knowledge mining, high-content screening and high-resolution mass spectrometry, to conduct a systematic screening in Zhongjing formulae, which is a large collection of TCM prescriptions with most abundant clinical evidences.

**Methods:**

Using Word2vec-based text learning, the correlations between 248 Zhongjing formulae and IBD typical symptoms were analyzed. Next, from the top three formulae with predicted relationship with IBD, TCM fractions were prepared and screened on a transgenic zebrafish IBD model for their therapeutic effects. Subsequently, the chemical compositions of the fraction hits were analyzed by mass spectrometry, and the major compounds were further studied for their anti-IBD effects and potential mechanisms.

**Results:**

Through knowledge mining, Peach Blossom Decoction, Pulsatilla Decoction, and Gegen Qinlian Decoction were predicted to be the three Zhongjing formulae mostly related to symptoms typical of IBD. Seventy-four fractions were prepared from the three formulae and screened in TNBS-induced zebrafish IBD model by high-content analysis, with the inhibition on the intestinal neutrophil accumulation and ROS level quantified as the screening criteria. Six herbal fractions showed significant effects on both pathological processes, which were subsequently analyzed by mass spectrometry to determine their chemical composition. Based on the major compounds identified by mass spectrometry, a second-round screen was conducted and six compounds (palmatine, daidzin, oroxyloside, chlorogenic acid, baicalin, aesculin) showed strong inhibitory effects on the intestinal inflammation phenotypes. The expression of multiple inflammatory factors, including *il1β*, *clcx8a*, *mmp* and *tnfα*, were increased in TNBS-treated fish, which were variously inhibited by the compounds, with aesculin showing the most potent effects. Moreover, aesculin and daidzin also upregulated *e-cadherin’s* expression.

**Conclusion:**

Taken together, we demonstrated the regulatory effects of several TCM formulae and their active compounds in the treatment of IBD, through a highly efficient research strategy, which can be applied in the discovery of effective TCM formulae and components in other diseases.

**Graphic abstract:**

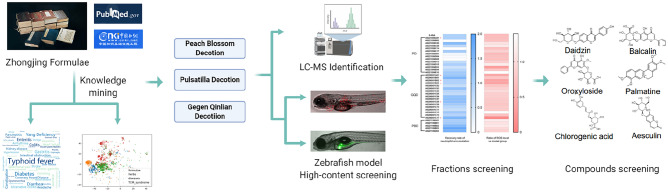

**Supplementary Information:**

The online version contains supplementary material available at 10.1186/s13020-021-00452-z.

## Highlights

- TCM formulae most related to IBD was identified by knowledge mining.
- Zebrafish-based high-content analysis was used to find effective TCM components in IBD.
- Aesculin and daidzin showed both anti-inflammation and epithelia protective effects.

## Introduction

Inflammatory bowel diseases (IBDs), which include Crohn’s disease and ulcerative colitis, are chronic relapsing gastrointestinal tract disorders characterized by diarrhea, abdominal cramping, fatigue, weight loss, and increased risk of developing colorectal cancers [1]. Despite previously considered as a disease of westernized countries, epidemiological studies in recent years identified rapidly accelerating incidences of IBDs in newly industrialized regions in Asia, Africa and South America, which make it a global health challenge [2]. The initiation and progression of IBDs are believed to be caused by the interactive results of multiple contributors, including environmental factors, genetics factors, infections and immune dysfunction. As a result, the clinical management of IBDs is challenging [3]. Aside from traditional treatment regimens such as aminosalicylates and corticosteroids, biological therapies have been actively explored in the past decade, such as monoclonal antibodies (mAbs) to tumor necrosis factor-α (TNF-α), interleukin (IL)-12 and IL-23. However, considering the complicate disease mechanism of IBDs, exploring new treatment options is still in high demand.

Traditional Chinese Medicine (TCM) has been used for millennia in China and many other Asian countries. However, despite numerous evidences were accumulated from clinical practices, most active compounds and targets of TCM remain ill-defined due to its complex chemical compositions, which severely hindered its interpretation by modern medicine. On the other hand, those knowledge recorded in classic TCM literatures may be overlooked. Because of the large amount and language obstacle, it is often difficult for nowadays clinicians or researchers to efficiently retrieve information from traditional TCM literatures, which were mostly written in ancient Chinese. Nevertheless, many information in these documents can be very useful. For example, the low heat resistance of the Nobel prize-winning discovery artemisinin was firstly inspired by an ancient TCM handbook [4]. IBD has been treated by TCM for thousands of years, although under the name of diverse clinical symptoms which are now included in the disease scope [5]. Possible mechanisms related to herbal medicines in IBD include maintenance of intestinal epithelial integrity, regulation of macrophage activation, modulation of immune response, and inhibition of TNF-α activity [6]. For example, curcumin alleviated the histopathologic changes in a trinitrobenzene sulfonic acid (TNBS)-induced mice IBD model, with suppressed CD4^+^ T-cell infiltration and NF-κB activation [7]. Nevertheless, the effects and mechanism of many botanical drugs in IBD are still not examined.

Aiming at the aforementioned challenges of TCM drug discovery, we combined the methods of computerized knowledge mining, high-content analysis, and high-resolution mass spectrometry (HRMS) in the current study. Firstly, systematic knowledge discovery of Zhongjing formulae, which was originally compiled by Zhang Zhongjing of Eastern Han Dynasty and regarded as the “progenitor of all TCM formulae”, was conducted using computerized text mining to determine the relationship between different prescriptions and clinical indications. Subsequently, high-content analysis was performed using a zebrafish IBD model, to screen for the effects of Zhongjing formulae in IBDs, the pipeline of which was further coupled with HPLC–MS, to identify active chemical compounds. The vertebrate animal model zebrafish is an ideal screening tool for high-content analysis, which has many advantages such as small body size, high fertility, rapid development, larvae transparency and low breeding cost. Moreover, zebrafish shares high conservation with mammals in most aspects, including genes regulating inflammatory signalings. TNBS is known to induce immunogenic reactions in the intestines of mice [8], and has also been used to establish IBD model in zebrafish [9, 10]. Since neutrophils accumulation in the epithelia exerted critical roles in modulating intestinal mucosal immune responses in IBD [11], and reactive oxygen species (ROS) is an essential mediator of mucosal injury [12], the intestinal accumulation of neutrophils and ROS were used as markers to evaluate the disease severity of IBD and the therapeutic effects in the fish model. Through the combination of modern scientific techniques, here we reported the identification of multiple active substances in the treatment of IBDs from ancient TCM literatures.

## Materials and methods

### Animal care ethics

All zebrafish experiments were conducted according to the guidelines of Animal Ethics Committee of the Laboratory Animal Center, Zhejiang University.

### Zebrafish husbandry

*Tg(Lyz:DsRED2)* transgenic fish [13] was obtained from the Laboratory Animal Center of Zhejiang University. Zebrafish were maintained following standard protocols [14]. E3 medium (0.29 g/l NaCl, 0.013 g/l KCl, 0.048 g/l CaCl_2_⋅2H_2_O, 0.082 g/l MgCl_2_⋅6H_2_O, pH 7.2) was used as the embryo medium. Embryos were obtained through natural spawning.

### Chemicals and reagents

TNBS (MB5547), 5-ASA (MB7539), and DCFH-DA (MA0219) were purchased from Dalian Meilun Biotechnology Company, China. 1-phenyl 2-thiourea (PTU) (P7629) and Ethyl 3-aminobenzoate methanesulfonate (Tricaine, E10521) were purchased from Sigma-Aldrich company of USA. Puerarin, 3ʹ-methoxypuerarin, daidzin, oroxindin, glycyrrhizic acid and esculin were purchased from Shanghai Yuanye Bio-Technology Co., Ltd (Shanghai, China). Palmatine chloride, chlorogenic acid, oroxyloside, fraxin and phellodendrine were purchased from Shanghai Winherb Medical Technology Co., Ltd (Shanghai, China). Berberine chloride was obtained from Dalian Meilun BiotechnologyCo., Ltd (Dalian, China). Baicalin was obtained from Shanghai Aladdin Bio-Chem Technology Co., Ltd (Shanghai, China). The purities of all compounds were no less than 98%. HPLC-grade acetonitrile, methanol and formic acid were from Merk (Darmstadt, Germany). Deionized water was prepared with an Elga PURELAB flex system (ELGA LabWater, UK).

### Knowledge mining

Forty ancient TCM books derived from or closely related to Zhongjing formulae were collected (Additional file 1: Table S1). A literature search was conducted on China National Knowledge Infrastructure and NCBI Pubmed, using “Shanghan”, “Zhongjing”, or the names of the 248 Zhongjing formulae as keywords, in the time window between 1999/01/01 and 2018/12/31. All articles were exported as the Refworks format. The word frequencies from the text of TCM books, or the titles and abstracts of articles were counted using Python (3.6). The lists of disease-related words and their frequencies were extracted, and the top 100 common words were visualized through word cloud generator in Python. Chinese phrases dictionaries were obtained from free online resources and imported into the program to automatically distinguish different types of phrases, e.g. herbs, formulae, syndromes, and diseases.

Text training was performed using Skip-gram models from Word2vec [15, 16]. Model files storing the words and their vectors were obtained as the output, through which the semantic distances between input and output words were calculated using cosine similarity. After descending the dimensionality, synonyms clustering can be visualized to assess the similarity among words. The resulted words vectors were visualized by t-distributed stochastic neighbor embedding. The training was conducted by Python with following parameters: size, 100; seed, 0; workers, 7. The set of word vectors was visualized by *t*-distributed stochastic neighbor embedding for dimensionality reduction.

### Fractions preparation and LC–MS analysis

The fractions were prepared as previously reported [17]. The fractions were dissolved in DMSO to 50 mg/ml for the stock solution for high content screening, and then diluted by water for LC–MS analysis. All the samples were centrifuged at 10,000 rpm for 20 min, and the supernatants were subject to LC–MS analysis. Acquity UPLC system (Waters, Milford, MA, USA) coupled with Triple TOF 5600 plus MS (AB SCIEX, Framingham, MA, USA) was employed for chemical identification. Chromatographic separation was carried out on Waters ACQUITY UPLC HSS T3 (100 mm × 2.1 mm i.d. 1.8 μm) at 40 °C with mobile phase A (0.1% formic acid–water) and mobile phase B(acetonitrile). The flow rate was 0.25 ml/min. Mass spectrometry analysis was performed in both positive and negative modes under following parameters: scan range, m/z 100–2000; ion source GS1, 50 psi; ion source GS2, 50 psi; curation gas (CUR), 35 psi; temperature, 600℃ for ESI^+^ and 550℃ for ESI^−^; ionspray voltage (IS), − 4.5 kV for ESI^−^ and 5.0 kV for ESI^+^.

### Zebrafish IBD modeling and drug treatment

For the construction of IBD model, 3 day post fertilization (dpf) zebrafish embryos were incubated in embryo medium supplemented with 75 μg/ml TNBS for 2 days, and 0.1% DMSO was used as a vehicle control. For drug protection, 2dpf embryos were incubated in 5-ASA (200 μg/ml), TCM fractions (50 μg/ml), or compounds (200 μM) for 24 h, and then replaced with TNBS treatment. In order to determine the concentration of TCM fractions and compounds, a dosage range was firstly estimated based on our previous experience [17–19]. Then the concentration was further adjusted according to their solubility in the embryo medium and their toxicity in zebrafish embryos. A concentration at which all fractions or compounds can be completely dissolved in the embryo medium, without causing obvious embryo toxicity was chosen for the screening. The toxicity assay of all compounds is shown in Additional file 2: Figure S1. The Phenylthiourea supplemented embryo medium was used to inhibit melanization as described before [20].

### High-throughput screening and data analysis

2dpf *Tg(Lyz:DsRED2)* transgenic embryos were dechorionated and dispensed in 96-well plate with 1 embryo/well. Respective drug protection was supplemented in 300 μl medium of each well at 2dpf and TNBS treatment was initiated at 3dpf. At 5dpf, embryos were incubated with 10 μM DCFH-DA for 30-min staining in the dark to label ROS. Then the fish embryos were anesthetized in 0.016% Tricaine with side facing up, and automatically imaged by ImageXpress Micro Confocal (Molecular Devices, US.). The number of neutrophils and ROS level were also analyzed by the ImageXpress analyzing tools with the intestinal region manually outlined. At least 5 embryos were examined for each treatment condition. Two sets of cutoff value were determined to evaluate the drug effects. For the quantification of neutrophils accumulation, “Recovery rate of neutrophils accumulation” was calculated by (N_model_ − N_drug_)/(N_model_ − N_control_). N represents for the number of neutrophils in the intestinal region, and a recovery rate > 0.6 was deemed to be effective. For ROS levels, average fluorescence intensity less than 70% of the value of the model group after drug protection was considered as effective.

### Histological analysis

5dpf Zebrafish embryos were fixed in 4% PFA at room temperature overnight, and were progressively dehydrated in ethanol. Embryos were embedded in paraffin blocks and were cut into 3 μm sections and stained with hematoxylin and eosin. At least three embryos were examined for each treatment condition.

### QPCR

Total RNA of zebrafish embryos was extracted by a RNA-Quick Purification Kit (RN001, ES Science), and then converted to single- strand cDNA with HiFiScript cDNA Synthesis Kit (CW2569M, CWBIO). Real-time PCR was performed using the two-step quantitative RT-PCR method with 2 X SYBR Green qPCR Mater Mix (B21202, Bimake). The sequences of all primers used in the study are listed in Additional file 1: Table S2.

### Statistical analysis

All data are presented as the mean ± the standard error of the mean (SEM). Differences between two groups were analyzed using the two-tailed Student’s t-test. Multiple group comparison was conducted by one-way ANOVA. An *p* value < 0.05 was considered statistically significant.

## Results

### Knowledge mining of Zhongjing formulae

Since Zhang Zhongjing created *Treatise on Febrile Diseases* at nearly 2000 years ago, it has been sorted out and edited numerous times by descendants. The formulae had also been clinically tested, and modified by following TCM doctors in many TCM handbooks, which were all referred as the classic Zhonging formulae. To avoid omissions, through careful literature review, we collected 40 ancient TCM books which are derived from or closely related to Zhongjing formulae, aside from the original book of *Treatise on Febrile Diseases* (Additional file 1: Table S1). Through word frequency analysis and vocabulary classification via Python, 248 TCM formulae were identified. A literature search was further conducted on China National Knowledge Infrastructure and NCBI Pubmed, using “Shanghan”, “Zhongjing”, or the names of the 248 Zhongjing formulae as keywords, in the time window between 1999/01/01 and 2018/12/31. In total, 79 016 articles from CNKI and 85 articles from PubMed were additionally included for knowledge mining.

**Fig. 1:**
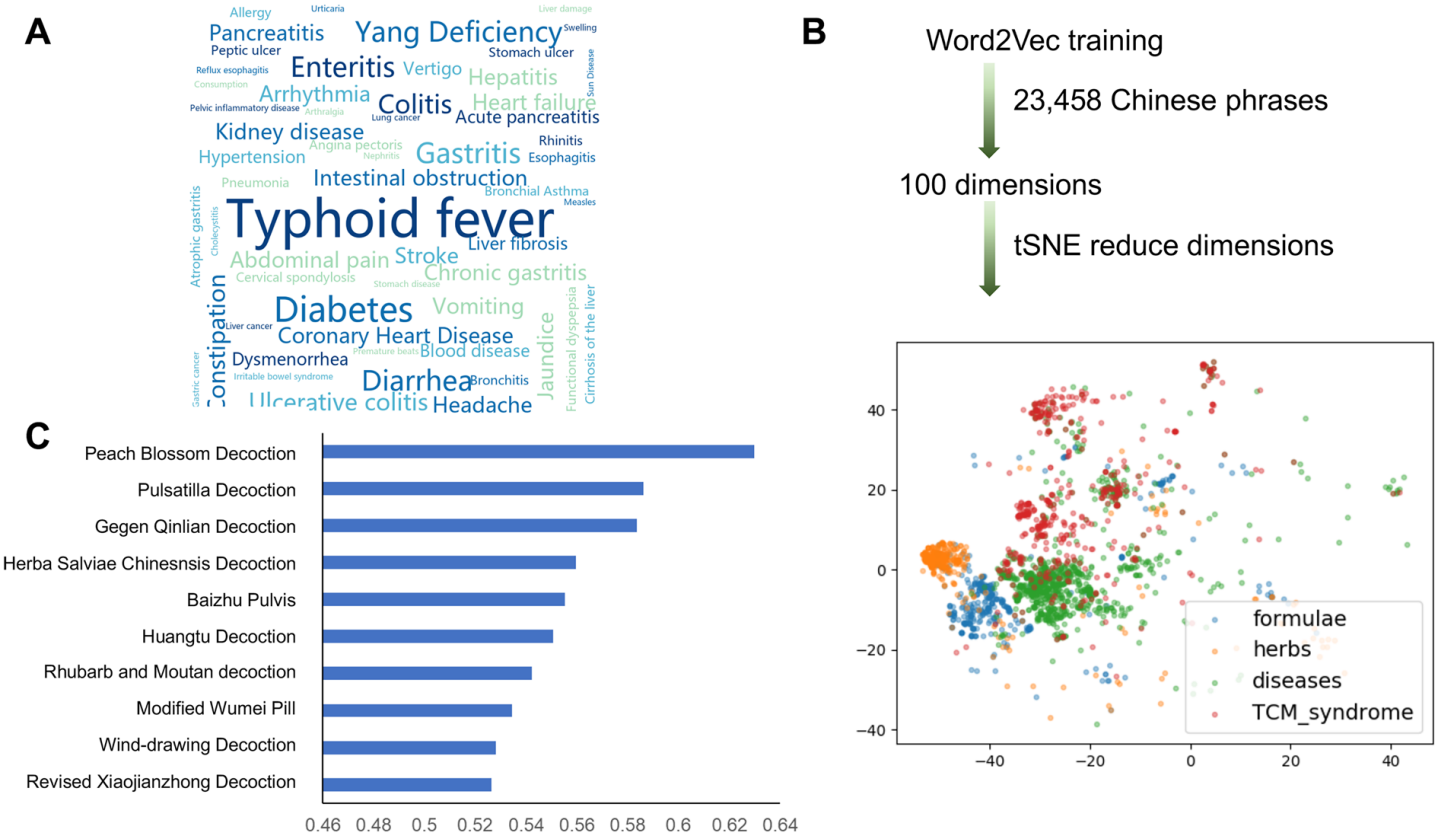
Knowledge mining of Zhongjing formulae. **A** Word cloud of top 100 common diseases-related phrases in included literatures; **B** Flow chart of Word2Vec based knowledge mining analysis; **C** Ten Zhongjing formulae most closely related to gastrointestinal inflammatory diseases

Knowledge mining was performed using the Word2vec model (Gensim, Python 3.6). Firstly, the word frequency of the text corpus was analyzed and the 100 most common words were retrieved (Fig. 1A). Noticeably, “enteritis”, “colitis”, “gastritis” were among the top hits of diseases or syndromes related to Zhongjing formulae, according to the frequency analysis results. Next, using the skip-gram model of Word2vec, enteritis related disease names or syndromes were used as the input value, and all the meaningful words and phrases in the context were automatically located and retrieved as the output value. The degree of correlation was calculated based on the semantic distances between the input and output words, with cosine value labeled as the marker. The higher the cosine value is, the closer the relationship was suggested. Interestingly, words in different categories tend to be clustered in different subgroups when visualizing by *t*-distributed stochastic neighbor embedding, which suggested the reliability of the text training (Fig. 1B). As a result, we identified ten Zhongjing formulae most closely related to gastrointestinal inflammatory diseases, as recommended by knowledge mining (Fig. 1C and Additional file 1: Table S3). The results of knowledge mining were further verified in the other direction. The names of Zhongjing formulae identified in the previous step were used as the input value, and the related symptoms or diseases in the context were predicted by the program. Consistently, a majority of symptoms related with these formulae are enteritis related, such as dysentery and diarrhea (Additional file 1: Table S4).

### TNBS induced typical IBD phenotypes in the zebrafish model

**Fig. 2:**
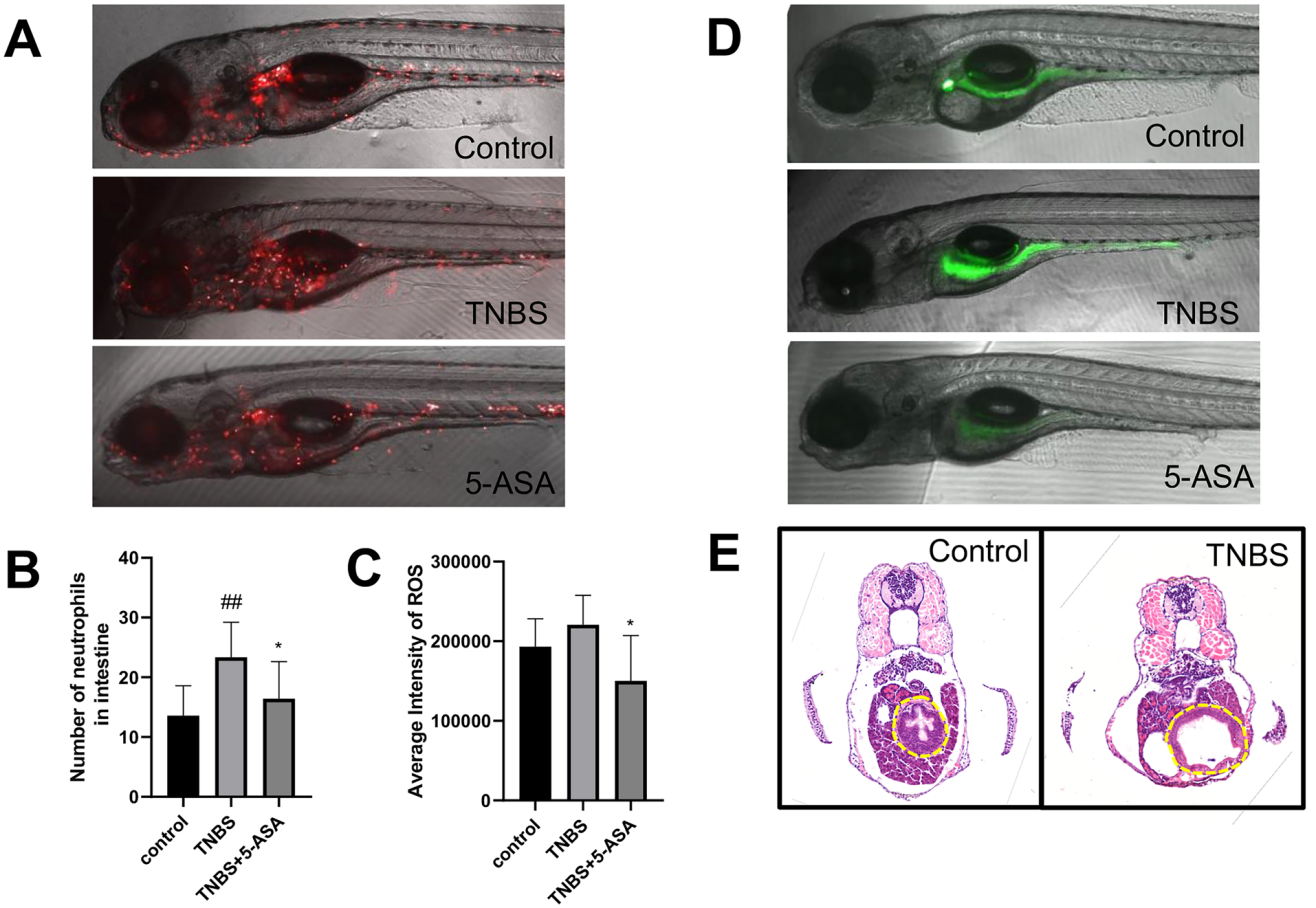
TNBS induced IBD phenotypes in zebrafish. **A**, **B** The intestinal distribution of red fluorescent-labeled neutrophils in control, TNBS and 5-ASA treated embryos; at least 6 embryos were examined for each group. **C**, **D** ROS staining (green signals) in control, TNBS and 5-ASA treated embryos; at least 5 embryos were examined for each group. **E** HE staining of embryo cross sections. The intestine regions were circled by yellow dotted lines. Scale bar: 100 μm. #compared with the control group; *compared with the model group; # or *, p < 0.05, ## or **, p < 0.01

Various IBD-like pathological changes were observed in TNBS-treated zebrafish in previous studies, such as intestinal morphological changes and increased neutrophils infiltration [9, 10]. Consistently, in our study, increased number of neutrophils was observed in the intestines of *Tg(Lyz:DsRED2)* transgenic fish, with fluorescein-labeled neutrophils under the lysozyme promoter [13], after 48 h stimulation in 75ug/ml TNBS solution from 72hpf (Fig. 2A, B). Besides, as accumulating evidences suggested that intestinal ROS can accelerate epithelial cell damage and initiate IBD [21], the level of oxidative stress was examined in the TNBS-treated zebrafish with the DCFH-DA probe, which is a fluorescence indicator of the formation of H_2_O_2_ or other ROS and RNS [22]. As previously reported, a considerable level of ROS signal was detected in the intestine of wildtype zebrafish embryos using DCFH-DA, possibly due to high ROS generation during normal intestinal development [23]. TNBS stimulation showed a tendency to further increase the level of gastrointestinal ROS, although with no statistical significance (Fig. 2C, D). Nevertheless, co-treatment of 5-aminosalicyclic acid (5-ASA), a standard therapeutic drug of IBDs, significantly decreased both neutrophils accumulation and the ROS signal in TNBS-treated embryos. Moreover, histological analysis detected reduced intestinal folds, enlarged gut lumen and disrupted epithelial layer in zebrafish larvae treated with TNBS, suggesting decreased peristalsis and impaired mucosal barrier (Fig. 2E). Taken together, these findings suggested that TNBS treatment is capable of inducing IBD phenotypes in zebrafish, and this model was used for subsequent screens in our study.

### High-throughput screening of the fractions of formulae hits

**Fig. 3:**
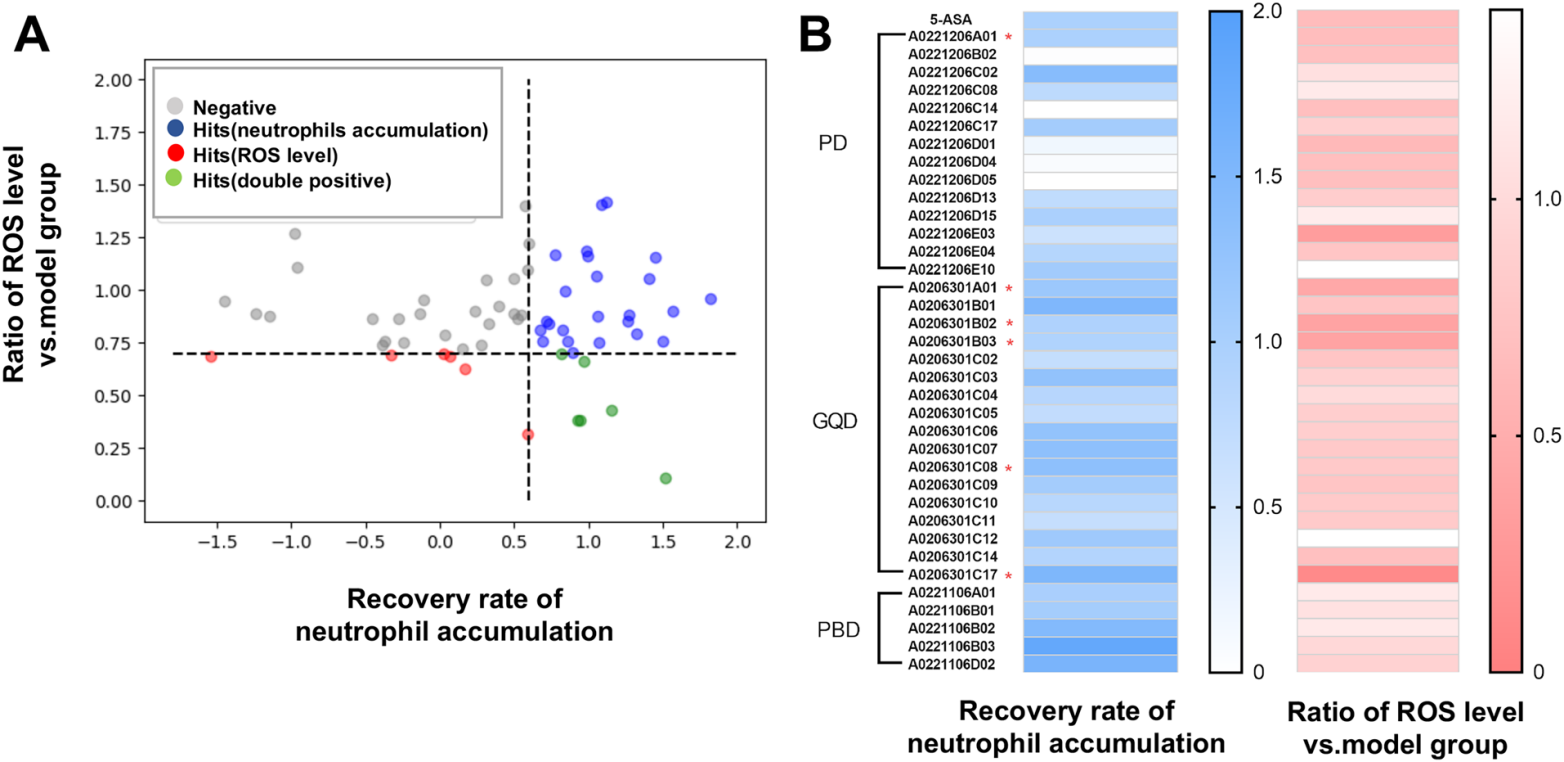
Results of high-content screening of Zhongjing formulae fractions. **A** Scatter plot showing the effects of 74 formulae on intestinal neutrophils accumulation and ROS. **B** Heat map showing the rescue levels of 36 formulae with significant effects at at least one screening criterion. The formulae with dual effects in regulating both neutrophil number and ROS are marked with a red asterisk

Based on the results of knowledge mining, three formulae with the highest likelihood to be related with IBD, as predicted by the Cosine value, were selected for further analysis, which were Peach Blossom Decoction (PBD), Pulsatilla Decoction (PD), and Gegen Qinlian Decoction (GQD) (Additional file 1: Table S2). Supportively, the therapeutic effects of these formulae in IBD and other enteritis were suggested by several other studies [24–26], but the active substances were still unclear. The fraction library of the three formulae was constructed as previously reported [17], which includes 74 fractions in total. Next, using the TNBS-induced zebrafish IBDs model, the therapeutic effects of these TCM fractions were screened. TCM fractions were supplemented to zebrafish embryos, at a dosage of 50 μg/mL, from 24 h before the TNBS treatment till the imaging analysis. The number of neutrophils and the level of ROS signals in the intestinal tract were used as the output indexes for efficacy evaluation. Using the performance of 5-ASA as a reference, two sets of cutoff values were used to facilitate efficient screening (see “Materials and methods” for details). As a result, 36 fractions were found to be effective for at least one marker, and six of them showed regulation on both markers (Fig. 3A, B). Interestingly, although five PBD fractions strongly inhibited the intestinal neutrophils accumulation, their effects on ROS signal were not significant. On the contrary, among the six fractions influence both neutrophils accumulation and ROS level, five fractions were derived from GQD. These findings suggested that the therapeutic effects of different TCM formulae rely on different pharmacological routes. In the current study, the six fractions with dual function on regulating neutrophils and ROS were included for the subsequent composition analysis and validation assay.

### Identification of main chemical compounds in active fractions by mass spectrometry

**Fig. 4:**
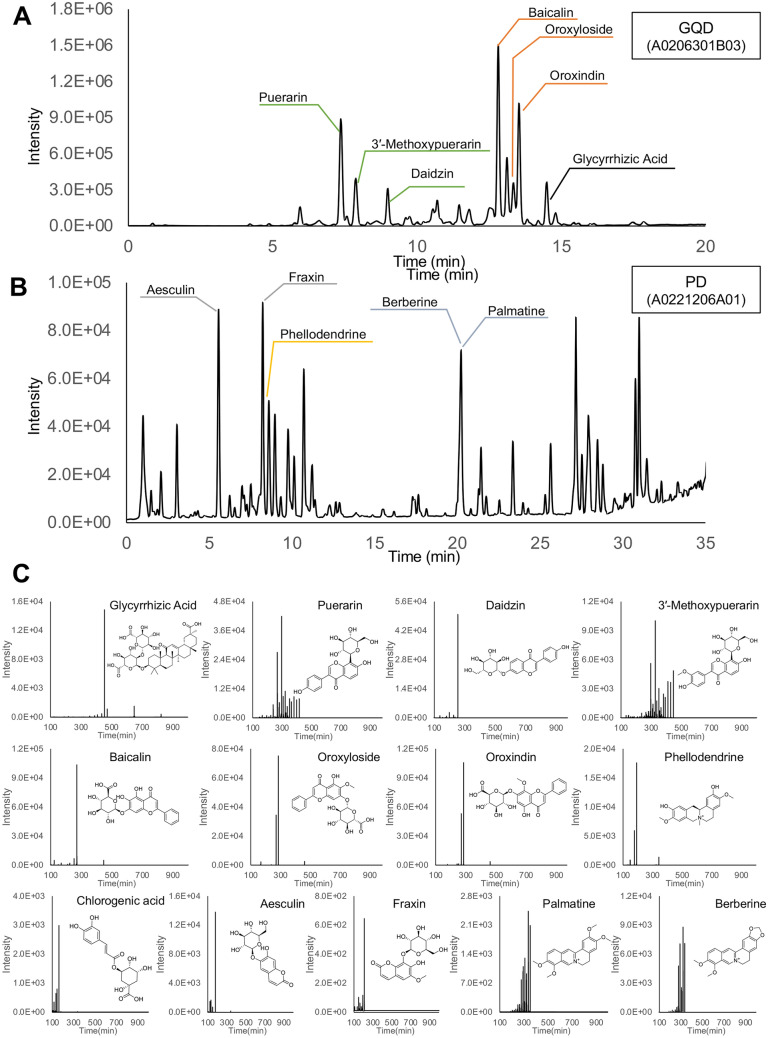
Component analysis of effective fractions identified in the screen. **A**, **B** Base peak chromatogram of fraction A0206301B03 of Gegen Qinlian Decoction (**A**) and fraction A0221206A01 of Pulsatilla Decoction (**B**) obtained by UPLC-Q-TOF in positive ion mode. The name of chemical compounds identified with reference standards are labeled. **C** Comparison of main chemical compounds with reference standards

Next, the chemical components in the six fractions which showed regulatory effects on both neutrophils accumulation and endogenous ROS level were analyzed by LC–MS in both negative and positive modes (Fig. 4 and Additional file 3: Figure S2). After examining the raw data, main peaks were identified in the fractions as major compounds. The molecular formulae of these compounds were obtained and the candidate structures were inferred from literatures and public databases. Based on the MS results, 7 compounds from GQD and 6 compounds from PD were selected for further identification, by comparisons with reference standards in terms of retention time and mass spectra. Among these compounds, 3 compounds were suggested to originate from the plant *Radix Puerariae*, 3 from *Scutellaria Baicalensis*, 2 from *Cortex Fraxini*, 2 from *Coptis Chinensis Franch*, 2 from *Phellodendron Chinense Schneid*, and 1 from *Glycyrrhiza Uralensis Fisch*. The chemical class of the potential active compounds includes flavonoids, saponins, alkaloids, phenolic acid and coumarins. The potential functions for these compounds by previous studies are summarized in Table 1, almost all of which were involved in the regulation of inflammatory processes.

**Table 1:** Summary of previously reported functions of compounds hits

Compound	Formula	Herbal origins	Suggested function
Baicalin	GQD	*Scutellaria Baicalensis*	Cardioprotection [45], enhancing glucose uptake and insulin sensitivity [46], anti-inflammation, anti-bacterial infection [47], anti-tumor [48], anti-oxidative stress, anti-allergy [49]
Daidzin	GQD	*Radix Puerariae*	Anti-tumor [50], anti-inflammation [51], anti-oxidative stress [52]
3ʹ-Methoxypuerarin	GQD	*Radix Puerariae*	Hepatoprotective [53]
Oroxindin	GQD	*Scutellaria Baicalensis*	Anti-inflammation, anti-tumor, anti-oxidative stress [54], anti-bacterial infection, anti-proliferation [55]
Glycyrrhizic acid	GQD	*Glycyrrhiza Uralensis Fisch*	Anti-viral infection [56], anti-inflammation [57], hepatoprotective [58], anti-cancer, anti‑ulcer, anti‑anaphylaxis [59]
Puerarin	GQD	*Radix Puerariae*	Vasodilation, cardio-protective [60], neuroprotection, anti-oxidation, anti-inflammation [61], anti-cancer [62], alleviating pain, promoting bone formation, inhibiting alcohol uptake [63], attenuating insulin resistance [60]
Oroxyloside	GQD	*Scutellaria Baicalensis*	Inhibiting angiogenesis [64], anti-cancer [65], hepatoprotective [66], anti-inflammation [29]
Esculin	PD	*Cortex Fraxini*	Hepatoprotective [67], anti-bacterial infection, anti-inflammation [68], anti‑allergy, skin protection [69], anti-thrombosis, anti-oxidative stress [70]
Fraxin	PD	*Cortex Fraxini*	Hepatoprotective, anti-oxidative stress [71], anti-inflammation [72]
Phellodendrine	PD	*Phellodendron Chinense Schneid*	Anti-oxidative stress, anti-inflammation, reducing blood pressure [73]
Palmatine	PD	*Coptis Chinensis Franch*	Anti-cancer, anti-oxidation, anti-inflammation, neuroprotection, anti-bacterial and virus infection, regulating blood lipids [74]
Berberine	PD	*Coptis Chinensis Franch*	Anti-microbial infection, anti-diabetis, anti-cancer, lipid-lowering, anti-diarrhea, antitrachoma, anti-oxidation, anti-inflammation, antiviral infection [75–77]
Chlorogenic acid	PD	*Phellodendron Chinense Schneid*	Anti-oxidative stress, anti-microbial infection, hepatoprotective, cardioprotective, anti-inflammation, antipyretxia, neuroprotection, anti-obesity, anti-hypertension, anti-oxidative stress, neuronal stimulation [78–81]

### Validation of active compounds with anti-IBD effects

**Fig. 5:**
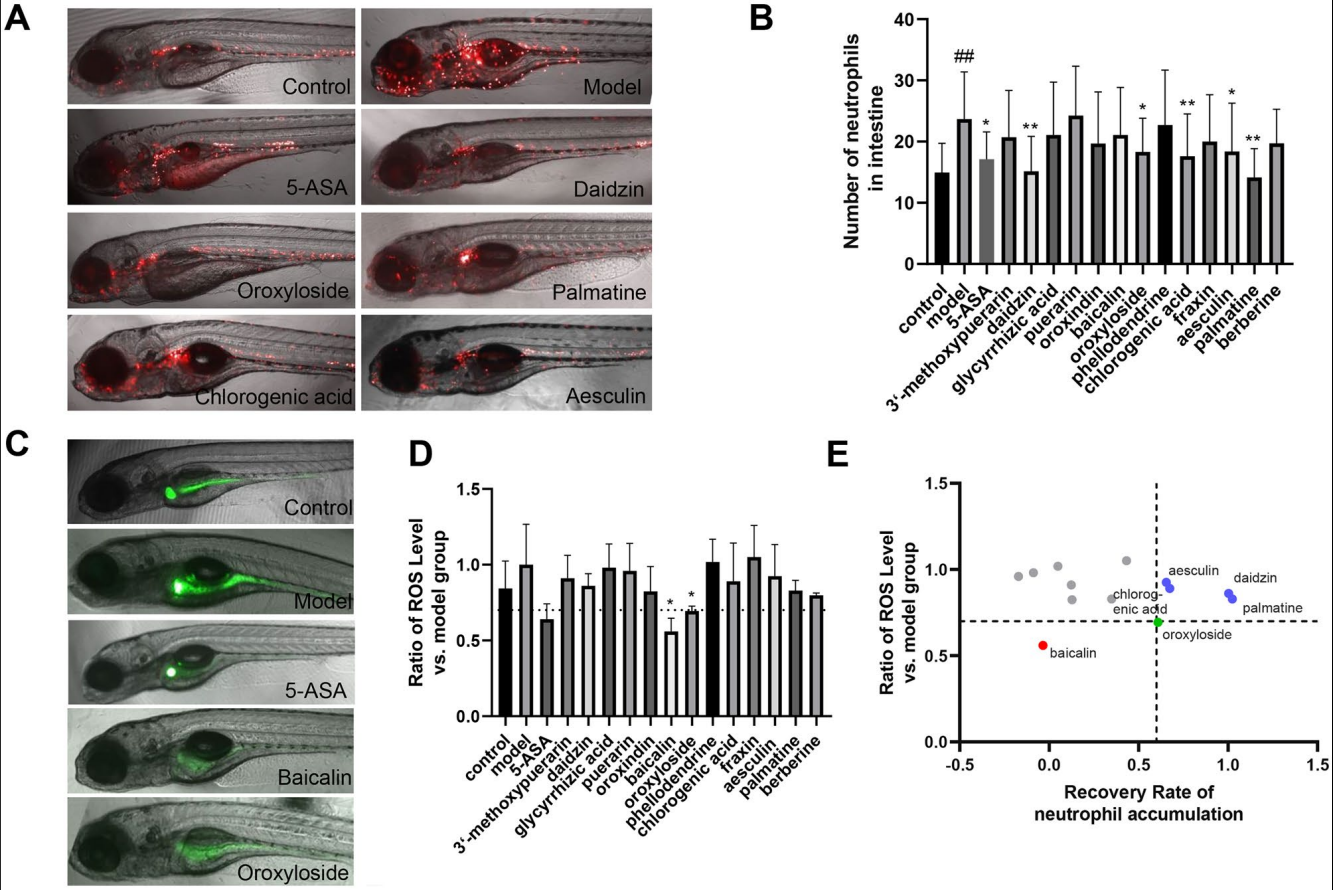
Results of high-content screening of Zhongjing formulae compounds. **A**, **B** Intestinal neutrophil accumulation in TNBS-stimulated embryos protected with different compounds; at least 10 embryos were examined for each group. **C**, **D** ROS level in embryos treated with different compounds; The dotted line in **D** marked the ratio of 0.7; at least 5 embryos were examined for each group. **E** Scatter plot summarized the effects of 13 compounds on intestinal neutrophils accumulation and ROS. Statistical comparisons were conducted between compound groups and model group. #compared with the control group; *compared with the model group; # or *, p < 0.05, ## or **, p < 0.01

Based on the results of HRMS, the above 13 compounds were included to further evaluate their regulatory effects on the development of intestinal inflammation in the TNBS-induced zebrafish IBDs model. Similar with the efficacy screening conducted for TCM fractions, the solution of each compound was supplemented to the embryo medium from 24 h before TNBS treatment till the end of the analysis, at a concentration of 200 μM. As a result, a total of 6 compounds showed protective effects against intestinal inflammation when using neutrophil accumulation or ROS level as readouts. Specifically, daidzin, palmatine, oroxyloside, chlorogenic acid, and aesculin significantly suppressed the accumulation of neutrophils in the intestinal tracts of fish IBD model, with daidzin and palmatine showing the most prominent effects (Fig. 5A, B). Besides, baicalin and oroxyloside are sufficient to downregulate the intestinal level of ROS when used alone (Fig. 5C, D). Oroxyloside, a flavonoid compound from the plant *Scutellaria Baicalensis*, was the only compound which displays moderate, but significant effects in the regulation of both neutrophil accumulation and oxidative stress. After literature review, we found more evidences in the inflammation regulation of the above compound hits from cell line or murine models [27–32], which suggests the reliability of our screening system. In addition, the regulation effect of daidzin in intestinal inflammation was novelly identified in our screen, which could be an interesting therapeutic candidate of IBD.

### The impacts of active compounds on the expression of inflammation and intestinal barrier markers

**Fig. 6:**
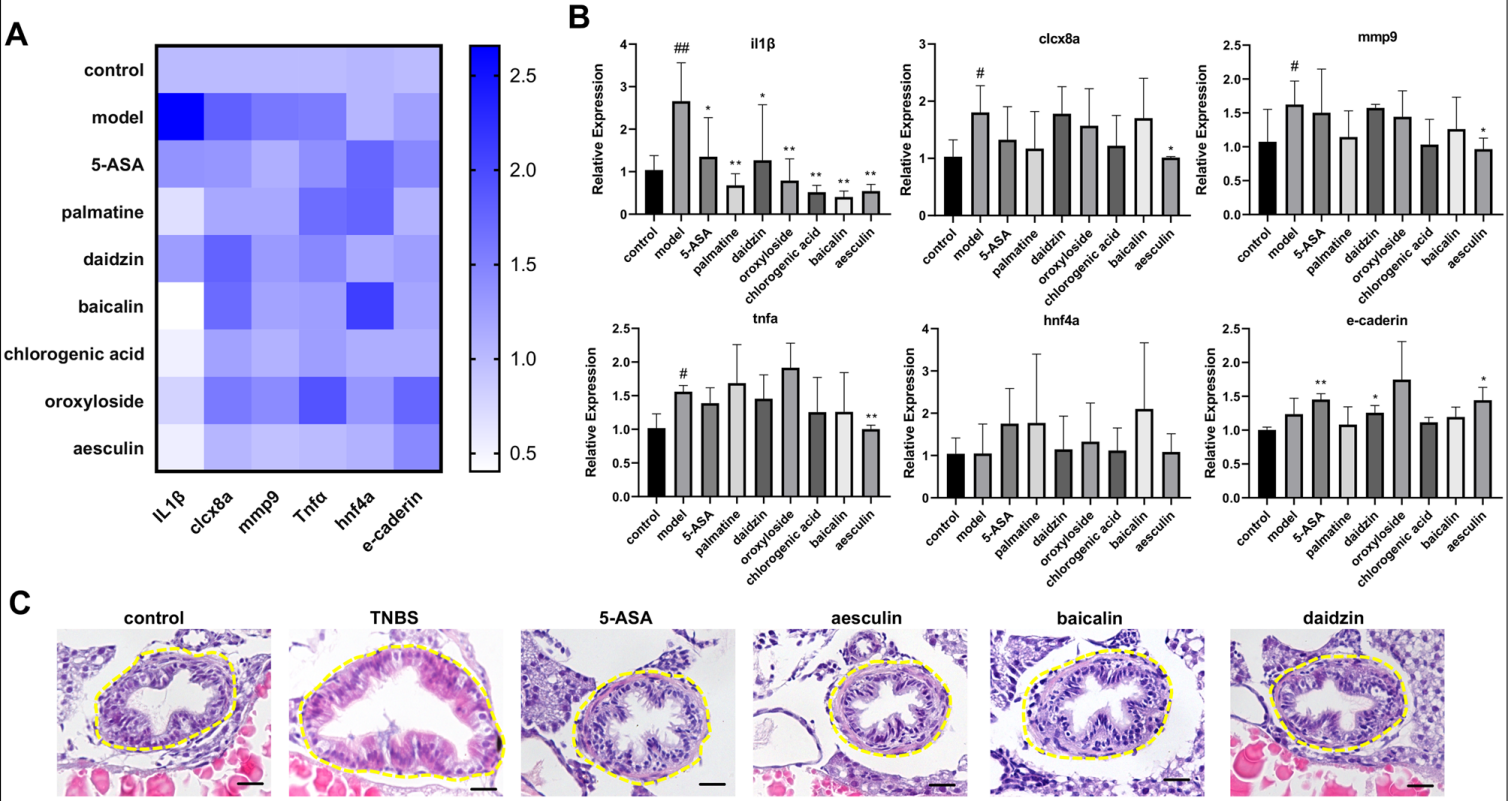
The impacts of active compounds on the expression of inflammation and intestinal barrier markers. **A**, **B** Heat map and histograms summarizing the expression levels of proinflammatory factors and epithelial barrier related genes. **C** Representative HE staining images of embryo treated with different drugs. The intestine regions were circled by yellow dotted lines. Scale bar: 20 μm. #compared with the control group; *compared with the model group; # or *, p < 0.05, ## or **, p < 0.01

Finally, we examined the potential influences of the Zhongjing formulae compounds on the endogenous expression of factors related to IBD development. After TNBS stimulation, the transcriptional level of multiple inflammatory related factors, including *il1β*, chemokine (C-X-C motif) ligand 8a (*cxcl8a*), matrix metallopeptidase 9(*mmp9*), and *tnfα* were all significantly increased (Fig. 6A, B). Noticeably, all the compounds displayed potent inhibitory effects on the expression of *il1β*, supporting their anti-inflammatory effects. Moreover, aesculin also significantly inhibited the mRNA levels of *cxcl8a*, *mmp9* and *tnf-α*. In order to analyze the effects of these compounds on intestinal barrier, the expression of adherens junction protein E-Cadherin and the epithelial transcription factor hepatocyte nuclear factor 4α (*hnf4α*) were also examined. Importantly, the transcriptional level of *e-cadherin* was upregulated by 5-ASA, daidzin and aesculin, suggesting that these agents may function through protecting the integrity of intestinal epithelial layer. We further inspected intestinal phenotypes directly by histological analysis. Compared with pathological alterations in the TNBS treated embryos, the intestinal morphology was largely maintained in all compound-treated groups (Fig. 6C).

## Discussion

Since the pathogenesis of IBD is still elusive, current treatment approaches of the disease are mostly symptomatically targeted, which aim to inhibit the progression of chronic intestinal inflammation. General inflammatory suppressant, such as corticosteroids and aminosalicylates, are routine IBD drugs. In recent years, the introduction of anti-TNF agents initiated a new era in IBD treatment. Infliximab and adalimumab can effectively induce clinical remission and mucosal healing in IBD patients [33]. However, one third of IBD patients failed to respond to anti-TNF treatment and 40% of initial responders developed intolerance over a year [34, 35]. Other biological immunomodulators, such as ustekinumab, an mAb against IL12/IL23, showed efficiency in moderately to severely active Crohn’s diseases, and brought clinical benefits in many patients who had failed anti-TNF treatment [36, 37]. Nevertheless, still more than 1/3 of the IBD patients showed no response to ustekinumab during the first year [38], and limited endoscopic remission was observed in ustekinumab treated patients after 24 weeks [36]. Therefore, ongoing drug development and mechanism research are needed for the management of IBD.

According to the TCM theory, specific combinations of herbs or other naturally-derived medicinal materials can generate cooperative or synergistic effects in treating particular diseases. Indeed, the therapeutic efficacies of many TCM formulae have been continuously testified by millennial clinical practices. However, after being modified by TCM doctors of many generations, the total amount of TCM formulae becomes very large, yet the descriptions of many formulae were only recorded in ancient languages, which made it difficult to review for nowadays researchers. On the other hand, dozens or even hundreds of compounds can be detected in even one herb, not to mention combinations of multiple herbs in a typical TCM formula. Thus, it is particularly challenging to identify the effective components. As a result, our understanding of TCM formulae was driven into a stagnant status for decades. Nevertheless, with the rapid development in modern techniques, re-examining the hidden secrets of TCM formulae may become possible.

Here, we innovatively combined the approaches of knowledge mining, high-content analysis and HRMS, to systematically identify effective TCM formulae and compounds in IBD treatment. Due to the large amount of healthcare data, computerized knowledge mining has been an intriguing area of medical research [39]. In order to unbiasedly distinguish TCM formulae related to IBD characteristic symptoms, both ancient and modern literatures of Zhongjing formulae, which is a large collection of TCM prescriptions with most abundant empirical evidences, were collected in our study, and the Word2vec algorithm was used to perform analogy retrieval and semantic relation retrieval [15]. As a result, PBD, PD and GQD were identified as the three formulae with the closest relationship with IBD. A previous randomized control trial demonstrated superior therapeutic effect of PBD in ulcerative colitis patients, compared with 5-ASA and glucocorticoid, with reduced levels of TNF-α and IL-8 [40]. PD administration attenuated the severity of colitis signs in mice induced by oxazolone or dextran sulfate sodium(DSS), which significantly reduced the secretion of pro-inflammatory cytokines and improved the colonic pathological manifestations [26, 41]. GQD oral administration was found to alleviate the severity of colitis in DSS-induced mice model, with reduced toll-like receptor 4 expression and NF-κB activation, as well as decreased level of pro-inflammatory cytokines [24]. Another study reported opposite changes of Notch signaling in acute and chronic ulcerative colitis mice models after DSS stimulation, whereas GQD was able to exhibit bidirectional regulation on Notch signaling [25]. Consistently, multiple fractions of the three TCM decoctions were found to exhibit significant regulatory effects in the zebrafish IBD model. Noticeably, despite being predicted as the top one TCM formula linked to IBD symptoms by knowledge mining, the fractions of PBD only showed significant effects in suppressing neutrophils recruitment, but not the endogenous ROS level, suggesting that its pharmacological mechanism may be different from PD and GQD. Other inflammation related markers and signaling pathways should be further examined to elaborate the molecular mechanism of these TCM decoctions in IBD treatment.

In the zebrafish IBD model, we further evaluated the regulatory effects of representative compounds. The zebrafish digestive system undergoes rapid development during early stages. In zebrafish embryos, a continuous gut tube formed at 2 to 3 dpf, and the mouth opened at about 4dpf. By 5dpf, the digestive tract can support the feeding and digestion processes, with the formation of many digestive organs functionally remseble their mammalian counterparts [42]. Based on previous experiences of others and our group [18, 19, 43], through soaking the zebrafish embryos in compounds supplemented growth medium, most chemical compounds can efficiently enter the blood circulation and digestive system of zebrafish embryos by direct skin penetration or absorption via mouthes or gills. Nevertheless, the rates of absorption and the pharmacodynamic characteristics of different drugs in zebrafish embryo are still unlcear, which may influence the quantitative evaluation of drug effects. Among the identified chemical compounds in our study, aesculin and daidzin are of particular interests to us. Aesculin is a coumarin compounds derived from *Cortex fraxini*, a main herb in PD. The protective effects of aesculin in DSS-induced colitis in mice have been reported previously, which may be mediated through the PPAR-γ and NF-κB pathway [30]. Consistently, aesculin rescued intestinal inflammation phenotype and restrained the upregulation of multiple inflammatory factors (*il1β*, *tnfα*, *clcx8a*, and *mmp9*) in our screening, suggesting its potent effect in inflammation regulation. Moreover, increased expression of *e-cadherin* was also observed in aesculin treated embryos. The adherens junctions of intestinal epithelial cells are formed mainly via E-cadherin, which is essential for the maintenance of intestinal barrier. As reported previously, knocking down *e-cadherin* led to severely aggravated symptoms of experimental colitis were in mice [44]. In addition, we also reported the protective effects of daidzin, an isoflavonoids compound derived from *Radix Puerariae*, in intestinal inflammation for the first time. Dramatically decreased neutrophil accumulation in the fish gut was observed in daidzin treated group, along with inhibited *il1β* expression and upregulated *e-cadherin* transcription. The positive regulation of aesculin and daidzin on *e-cadherin’s* expression, on top of their anti-intestinal inflammation effects, making them inviting IBD drug candidates.

Limitations remain existed in our study. For the current attempt of high-content screening, we only used two sets of screening outputs, intestinal neutrophils accumulation and ROS level, which may result in the omission of formulae or compounds that do not function through these two pathological processes. Some modifications can be made in future studies. For example, by using intestinal epithelial cells labeled transgenic fish lines, the intestinal morphological changes can be directly observed and applied as a golden standard for the screening. Besides, other fluorescent probes for key proinflammatory factors can also be explored in the screen, to identify compound with specific regulatory effects on a particular target protein.

## Conclusion

Aiming at the drug development needs in IBD treatment, we performed a systematic and unbiased screening in Zhongjing formulae via combining the techniques of knowledge mining, live embryo-based high-content analysis, and high-resolution mass spectrometry. The regulatory effects of TCM formulae fractions and representative compounds were demonstrated in our study, with aesculin and daidzin being particularly interesting IBD drug candidates. Future studies are warranted to further investigate the downstream targets and pharmacological mechanism of these TCM formulae and compounds in IBD treatment. The research strategy in our study can also be applied in the discovery of effective TCM formulae and components in other diseases.

## Supplementary Information

**Additional file 1: Table S1.** List of ancient Zhongjing Formula related ancient TCM books included for the knowledge mining. **Table S2.** List of QPCR primers used in this study. **Table S3.** List of top ranking Zhongjing formulae related to inflammatory bowel diseases based on the results of knowledge mining. **Table S4.** List of top ranking syndromes related to the IBD-related Zhongjing formulae based on the results of knowledge mining.

**Additional file 2: Figure S1.** Toxicity assay of all compounds in the screening.

**Additional file 3: Figure S2.** Base peak chromatogram of all fraction hits obtained by UPLC-Q-TOF in negative (NEG) or positive(POS) ion modes.

## Data Availability

The datasets used during the current study are available from the corresponding author on reasonable request.

## Abbreviations

Cxcl8a: Chemokine (C-X-C motif) ligand 8a
GQD: Gegen Qinlian Decoction
hnf4α: Hepatocyte nuclear factor 4α
HRMS: High-resolution mass spectrometry
IBD: Inflammatory bowel diseases
IL: Interleukin
mAbs: Monoclonal antibodies
mmp9: Matrix metallopeptidase 9
PBD: Peach Blossom Decoction
PD: Pulsatilla Decoction
ROS: Reactive oxygen species
TCM: Traditional Chinese medicine
TNBS: Trinitrobenzene sulfonic acid
TNF-α: Tumor necrosis factor-α
5-ASA: 5-Aminosalicyclic acid
