# Supplementary material for: Identification of anti-inflammatory compounds from Zhongjing formulae by knowledge mining and high-content screening in a zebrafish model of inflammatory bowel diseases
Source: Chin Med. 2021 May 31;16:42. doi: 10.1186/s13020-021-00452-z (PMC8166029; doi:10.1186/s13020-021-00452-z)
Supplement: Supplementary file 1 — Additional file 1: Table S1. List of ancient Zhongjing Formula related ancient TCM books included for the knowledge mining. Table S2. List of QPCR primers used in this study. Table S3. List of top ranking Zhongjing formulae related to inflammatory bowel diseases based on the results of knowledge mining. Table S4. List of top ranking syndromes related to the IBD-related Zhongjing formulae based on the results of knowledge mining. [file 13020_2021_452_MOESM1_ESM.docx]

**Title: Identification of anti-inflammatory compounds from Zhongjing Formulae by knowledge mining and high-content screening in a zebrafish model of inflammatory bowel diseases**

**Table S1: List of ancient Zhongjing Formula related ancient TCM books included for the knowledge mining.**

| NO. | Book | Author | Dynasty |
| --- | --- | --- | --- |
| 1 | Ao Shi Shang Han Jin Jing Lu | Dong Ben | Yuan |
| 2 | Ding Zheng Zhong Jing Quan Shu Jin Gui Yao Lue Zhu | Unknown | Unknown |
| 3 | Gao Zhu Jin Gui Yao Lue | Gao Xue Shan | Qing |
| 4 | He Jian Shang Han Xin Yao | Liu Hong | Jin |
| 5 | Jin Gui Xuan Jie | Unknown | Unknown |
| 6 | Jin Gui Yao Lue Fang Lun | Wang Zhu Lu | Song |
| 7 | Jin Gui Yao Lue Qian Zhu | Chen Xiu Yuan | Han |
| 8 | Jin Gui Yao Lue Xin Dian | You Yi | Qing |
| 9 | Jin Gui Yi | You Yi | Qing |
| 10 | Jin Gui Yu Han Jing Dong Zhu | Zhou Yang Jun | Qing |
| 11 | Jin Gui Yu Han Yao Lue Ji Yi | Yuan Jian | Qing |
| 12 | Jin Gui Yu Han Yao Lue Shu Yi | Wang Zhu Lu | Song |
| 13 | Jing Fang Shi Yan Lu | Cao Ying Fu | Qing |
| 14 | Lei Zheng Huo Ren Shu | Zhu Gong | Song |
| 15 | Shang Han Bai Zheng Ge | Xu Shu Wei | Song |
| 16 | Shang Han Biao Ben Xin Fa Lei Cui | Liu Wan Su | Jin |
| 17 | Shang Han Bu Li | Zhou Xue Hai | Qing |
| 18 | Shang Han Da Bai | Gao Shi Shi | Qing |
| 19 | Shang Han Fa Wei Lun | Xu Shu Wei | Song |
| 20 | Shang Han Fa Zu | Ren Yue An | Song |
| 21 | Shang Han Fu Yi | Ke Qin | Qing |
| 22 | Shang Han Guan Zhu Ji | You Yi | Qing |
| 23 | Shang Han Heng Lun | Zheng Shou Quan | Qing |
| 24 | Shang Han Jie Jue | Yan Ze An | Qing |
| 25 | Shang Han Jiu Shi Lun | Xu Shu Wei | Song |
| 26 | Shang Han Kuo Yao | Li Zhong Zi | Ming |
| 27 | Shang Han Liu Shu | Tao Hua | Ming |
| 28 | Treatise on Febrile Diseases | Zhang Zhong Jing | Han |
| 29 | Shang Han Ming Li Lun | Cheng Wu Ji | Jin |
| 30 | Shang Han She Jian | Zhang Deng | Qing |
| 31 | Shang Han Shuo Yi | Huang Yuan Yu | Qing |
| 32 | Shang Han Xin Fa Yao Jue | Wu Qian | Qing |
| 33 | Shang Han Xuan Jie | Huang Yuan Yu | Qing |
| 34 | Shang Han Xun Yuan | Lv Zhen Ming | Qing |
| 35 | Shang Han Yi Jue Chuan Jie | Chen Nian Zu | Qing |
| 36 | Shang Han Zhi Ge | Unknown | Jin |
| 37 | Shang Han Zhi Zhang | Wu Kun An | Qing |
| 38 | Shang Han Zong Bing Lun | Pang An Shi | Song |
| 39 | Ancient Chinese Medicine In Circular Movement | Peng Zi Yi | Qing |
| 40 | Zhong Jing Shang Han Bu Wang Lun | Guo Yong | Song |

**Table S2: List of QPCR primers used in this study.**

| Gene | Forward (5’-3’) | Reverse (3’-5’) |
| --- | --- | --- |
| *ef1a*  (internal reference) | AGAAGGCTGCCAAGACCAAG | AGAGGTTGGGAAGAACACGC |
| *il1β* | TGTGTGTTTGGGAATCTCCA | CTGATAAACCAACCGGGACA |
| *clcx8a* | TGTTTTCCTGGCATTTCTGACC | TTTACAGTGTGGGCTTGGAGGG |
| *mmp9* | CATTAAAGATGCCCTGATGTATCCC | AGTGGTGGTCCGTGGTTGAG |
| *tnfα* | ACCAGGCCTTTTCTTCAGGT | TTTGCCTCCGTAGGATTCAG |
| *hnf4a* | TACTAGGAGCTGCCAAACGC | TTCTTACAGCCACACGGCTC |
| *e-cadherin*(*cdh1*) | GGCTTGTGTAACAACTGTGGG | GCCACTGTGAAGGTGATTTCG |

**Table S3: List of top ranking Zhongjing formulae related to inflammatory bowel diseases based on the results of knowledge mining.**

| NO. | Formulae | Degree of correlation |
| --- | --- | --- |
| 1 | Peach Blossom Decoction | 0.629894 |
| 2 | Pulsatilla Decoction | 0.586124 |
| 3 | Gegen Qinlian Decoction | 0.583605 |
| 4 | Herba Salviae Chinesnsis Decoction | 0.559713 |
| 5 | Baizhu Pulvis | 0.555489 |
| 6 | Huangtu Decoction | 0.550651 |
| 7 | Rhubarb and Moutan decoction | 0.542482 |
| 8 | Modified Wumei Pill | 0.534484 |
| 9 | Wind-drawing Decoction | 0.528301 |
| 10 | Revised Xiaojianzhong Decoction | 0.526435 |

**Table S4: List of top ranking syndromes related to the IBD-related Zhongjing formulae based on the results of knowledge mining.**

| Syndromes | Degree of Correlation |
| --- | --- |
| Summer fever | 0.667794 |
| Dysentery | 0.665368 |
| Infantile diarrhea | 0.662441 |
| Intestinal damp-heat syndrome | 0.641941 |
| Acute renal failure | 0.613998 |
| Syndrome of cold invading the lungs | 0.611669 |
| Dampness and heat in the large intestine | 0.604703 |
| Swollen cheek | 0.597434 |
| Enduring diarrhea | 0.590709 |
| Anorexia | 0.58808 |
| Chickenpox | 0.587314 |
| Dyspepsia caused by excessive eating or improper diet | 0.580799 |
| Epidemic cold | 0.577655 |
| Syndrome of deficiency of spleen qi and stomach qi | 0.576509 |
| Prolapse of the anus | 0.576467 |
| Pneumoconiosis | 0.568407 |
| Blood in the stool | 0.55868 |
| Cold syndrome of qi stagnation | 0.554213 |
| Lung yin deficiency syndrome | 0.55345 |
| Asthenia of spleen and stagnation of wetness-evil | 0.545396 |
| Ischemic Stroke | 0.544642 |
| Bowel Cancer | 0.538095 |
| Stomach slowing | 0.535508 |
| Pertussis | 0.530234 |
| syndrome of liver effulgence and spleen deficiency | 0.529444 |
| Constipation | 0.527483 |
| Syndrome of stagnation and heat of liver and stomach | 0.525895 |
| Pulmonary fever | 0.519899 |
| Bladder cancer | 0.518689 |
| Gallstone | 0.516827 |
| Hepatic fire attacking lung | 0.516213 |
| Stone gonorrhea | 0.510516 |
| Heat gonorrhea | 0.509491 |
| Deficiency of spleen-yin | 0.508845 |
| Measles | 0.50834 |
| Intestinal carbuncle | 0.507322 |
| Epigastric | 0.505687 |
| Deficiency of spleen qi and lung qi | 0.505503 |
| Cold | 0.501165 |
