# Supplementary material for: Identification of anti-inflammatory compounds from Zhongjing formulae by knowledge mining and high-content screening in a zebrafish model of inflammatory bowel diseases
Source: Chin Med. 2021 May 31;16:42. doi: 10.1186/s13020-021-00452-z (PMC8166029; doi:10.1186/s13020-021-00452-z)
Supplement: Supplementary file 2 — Additional file 2: Figure S1. Toxicity assay of all compounds in the screening. [file 13020_2021_452_MOESM2_ESM.pdf]

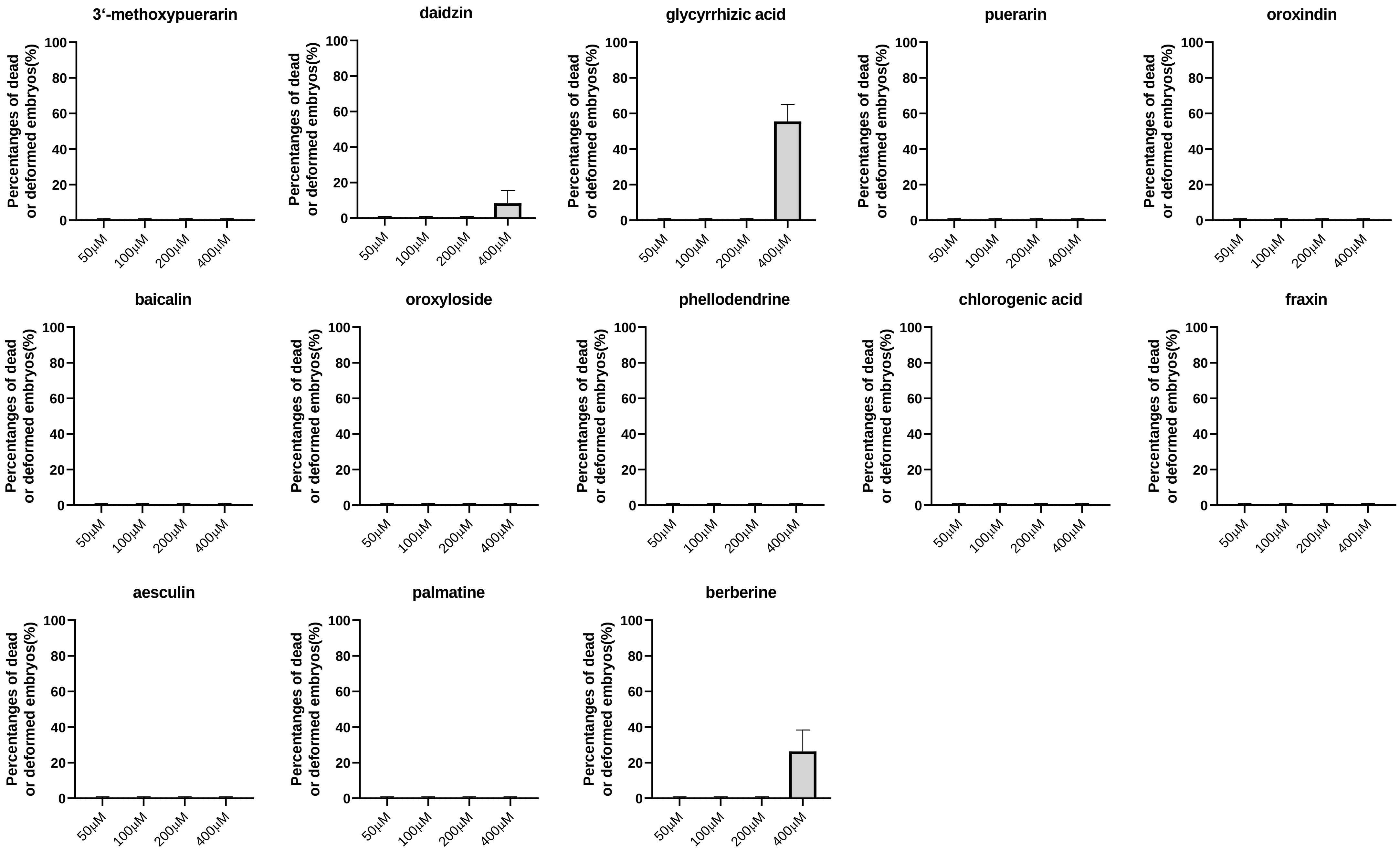
3‘-methoxypuerarin
daidzin
glycyrrhizic acid
puerarin
oroxindin
baicalin
oroxyloside
phellodendrine
chlorogenic acid
fraxin
aesculin
palmatine
berberine
Percentanges of dead
or deformed embryos(%)
100
80
60
40
20
0
50μM
100μM
200μM
400μM
