# Supplementary figures and images for: Identification of anti-inflammatory compounds from Zhongjing formulae by knowledge mining and high-content screening in a zebrafish model of inflammatory bowel diseases
Source: Chin Med. 2021 May 31;16:42. doi: 10.1186/s13020-021-00452-z (PMC8166029; doi:10.1186/s13020-021-00452-z)

A0206301A01  
-NEG

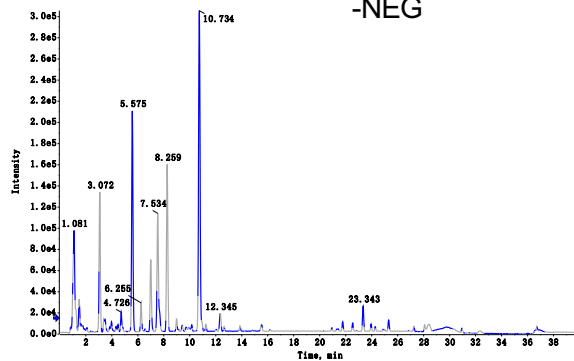

A0206301A01  
-POS

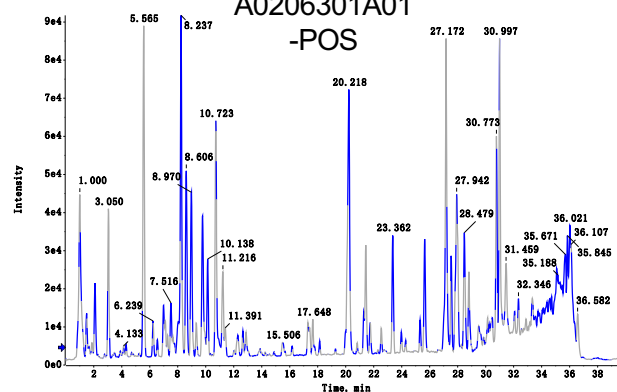

A0206301B02  
-NEG

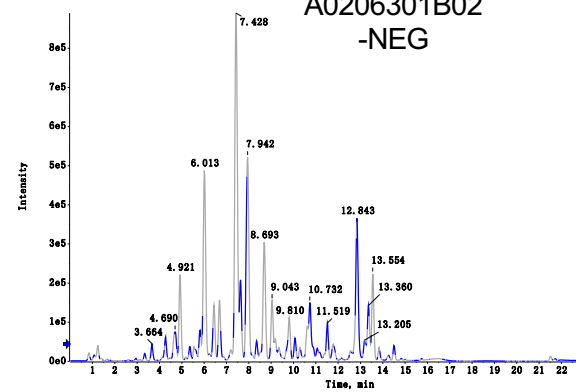

A0206301B02  
-POS

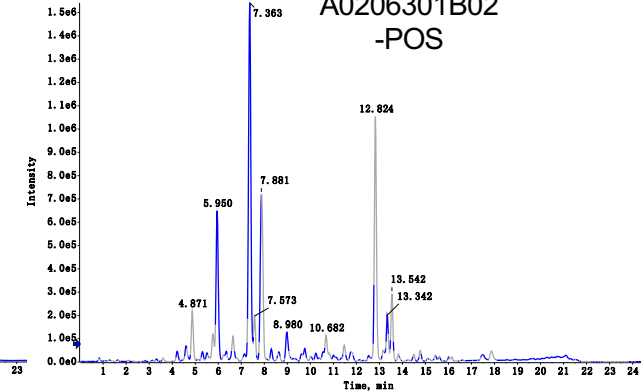

A0206301B03  
-NEG

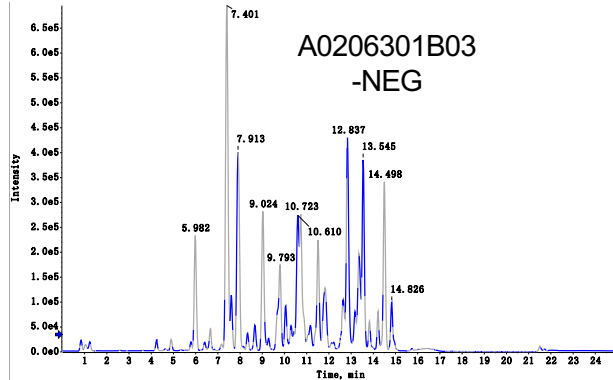

A0206301B03  
-POS

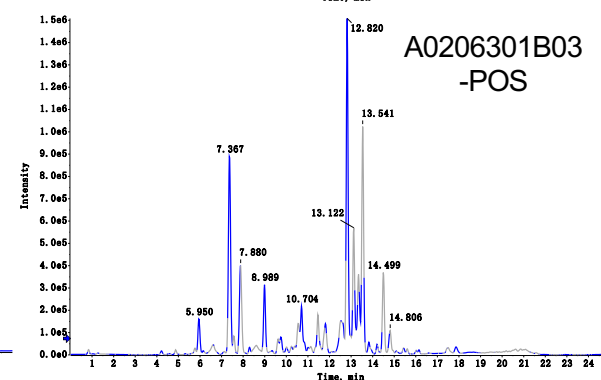

A0206301C08  
-NEG

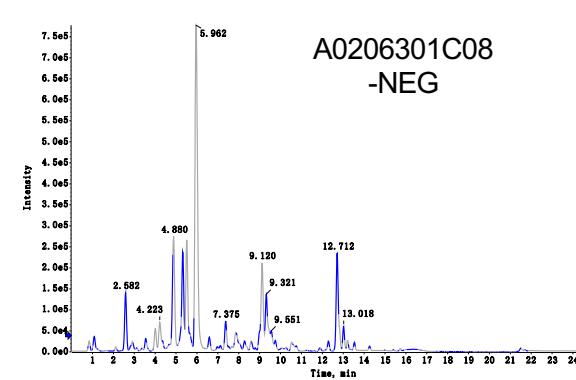

A0206301C08  
-POS

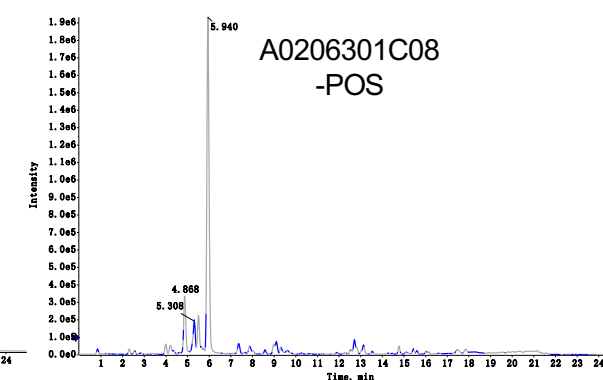

A0206301C17  
-NEG

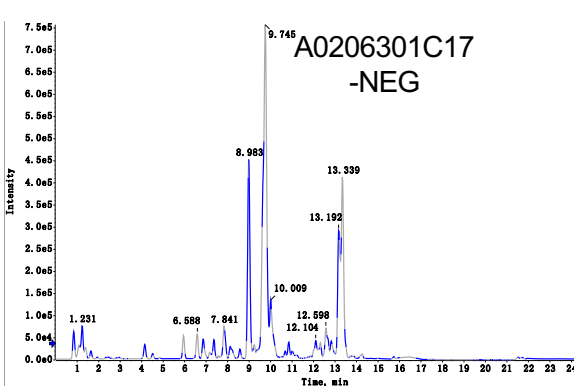

A0206301C17  
-POS

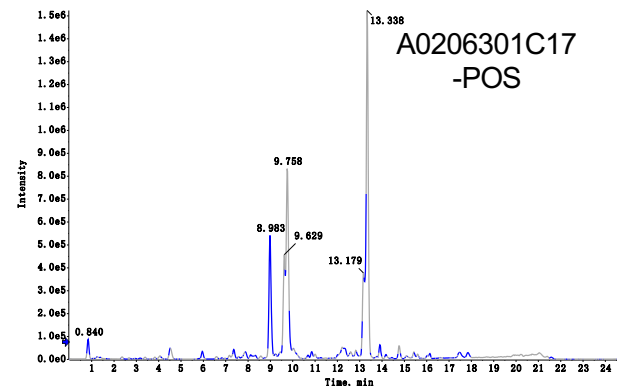

A0221206A01  
-NEG

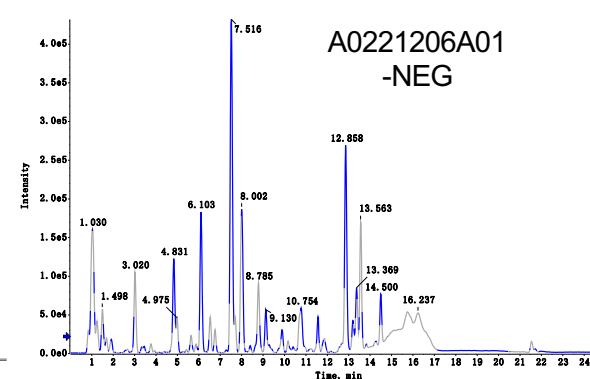

A0221206A01  
-POS

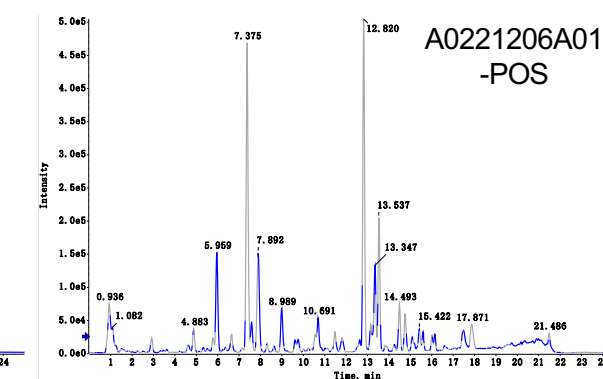

Supplement: Supplementary file 3 — Additional file 3: Figure S2. Base peak chromatogram of all fraction hits obtained by UPLC-Q-TOF in negative (NEG) or positive(POS) ion modes. [file 13020_2021_452_MOESM3_ESM.pdf]
